# Supplementary material for: Spatial disparities and multilevel determinants of childhood diarrhea in Mozambique: Evidence from the 2022–2023 Demographic and Health Survey (DHS)
Source: PLoS One. 2026 May 26;21(5):e0330240. doi: 10.1371/journal.pone.0330240 (PMC13210273; doi:10.1371/journal.pone.0330240)
Supplement: Attachment — Submitted filename: Response to Reviewers 2.pdf [file pone.0330240.s001.pdf]

Response to Reviewers' Comments

Subject: Response to Data Availability Statement Request

Dear Editor,

Thank you for your request regarding the Data Availability Statement. We confirm that a complete Data Availability Statement has been provided in the submission form. The statement specifies that the Mozambique Demographic and Health Survey (DHS) 2022–2023 data are available from the DHS Program upon registration and approval, and that the administrative boundary shapefiles were obtained from the Humanitarian Data Exchange (HDX) and are openly accessible.

We trust that this satisfies the journal's data availability requirements.

Line / Section	Reviewer Comment	Response
**General Comments**	This manuscript offers a valuable and timely contribution… Minor revisions are needed to improve clarity and presentation—specifically in reference formatting, language polish, and deeper discussion of spatial findings and study limitations.	We sincerely thank the reviewer for the positive and constructive feedback. We have revised the manuscript to enhance clarity, improve language quality, correct reference formatting, and expand the discussion on spatial findings and study limitations.
**Title, Line 2**	Change "Among" to "among."	Revised as suggested.
**Abstract, Line 24–25**	"with its burden varying across regions" → "with regional variations in its burden."	Revised for clarity.
**Abstract, Line 29**	"assess diarrhea prevalence and determinants" → "examine the prevalence and determinants of diarrhea."	Revised as suggested.
**Abstract, Line 33**	Re-write results for clarity.	Reworded to clearly present prevalence, spatial clusters, and multilevel determinants.
**Introduction, Line 53**	"Disease" → "disease."	Corrected.
**Introduction, Line 54**	"But, it is both preventable and treatable" → "However, it is preventable and treatable."	Revised.
**Introduction, Line 61**	"teta" → "Tete."	Corrected.

**Introduction, Line 77–78**	"Regarding to SDG goal three the Mozambique planned to reduce under five children mortality by 25/1000" → "In alignment with SDG Goal 3, Mozambique has committed to reducing under-five mortality to 25 per 1,000 live births (18, 19)."	Revised.
**Introduction, Line 55–57**	Clarify 444,000 deaths vs. previous estimates (390,000 in 2021).	"According to WHO and UNICEF estimates (2024–2025), ~444,000 children under five die globally each year [1,2]. Of these, diarrhea accounted for ~340,000–390,000 deaths in 2021, indicating modest reductions over time but highlighting ongoing challenges."
**Introduction, Line 70–72**	Group risk factors as individual-level, household-level, environmental.	Rewritten: "Childhood diarrhea is influenced by factors at multiple levels, including individual (age, breastfeeding, vaccination, nutrition), household (size, caregiving, maternal age and education), and environmental (water source, sanitation, socioeconomic status)."
**Introduction, Line 83–86**	Re-write study objective.	Revised: "This study aims to assess the spatial distribution and multilevel determinants of diarrhea among children under five in Mozambique using recent DHS data and spatial modeling tools such as SaTScan and ArcGIS."
**Introduction, Context Comment**	Lack of contextual information about Mozambique and prior interventions.	Added context on Mozambique's actions: rotavirus and oral polio vaccination, WASH improvements, IMCI guideline training, promotion of exclusive breastfeeding, zinc supplementation, and community-based education. Regional disparities persist.
**Methods, Line 89**	Arrange subheadings logically.	Methods reorganized into: Study design and setting, Data source, Study population, Outcome variable, Explanatory variables, Spatial analysis, Multilevel analysis, Statistical analysis, Ethical considerations.

**Methods, Line 92**	"the bulk of whom live in rural areas" → "with the majority residing in rural areas (23–25)."	Revised.
**Methods, Line 99**	Clarify sample size statement.	"The DHS sampled a nationally representative set of approximately 9,799 households."
**Methods, Line 103–104**	"The second stage involves updating household listings" → "In the second stage, household listings were updated."	Corrected tense.
**Methods, Line 108**	Clarify final analytic sample.	"After excluding children with missing diarrhea data and incomplete household information, the final analytic sample included 9,799 children under five."
**Methods, Line 108–109**	"concentrating on the past two weeks of diarrhea history before to the survey" → "in the two weeks prior to the survey."	Corrected.
**Methods, Line 183–184**	Define MOR, ICC, PCV.	Definitions and formulas added for clarity.
**Methods, Spatial Analysis Comment**	Is spatial analysis at individual or cluster level?	Conducted at DHS cluster level. Cluster-level GPS coordinates used; individual coordinates unavailable for privacy. Aggregated cluster-level prevalence or residuals used for hotspot detection (Gi* Z-score) and spatial autocorrelation (Moran's I). Clarified in Methods.
**Methods, Weights Comment**	Were survey weights accounted for?	All analyses, including multilevel and spatial analyses, accounted for DHS complex survey design. Estimates and analyses used sampling weights and accounted for clustering and stratification.
**Methods, Figure 6 Comment**	Forest plot unnecessary; horizontal axis not logarithmic.	Figure 6 removed as it was not essential.
**Methods, Figures 1 & 2 Comment**	Take up space; move to supplementary materials.	Figures 1 and 2 moved to supplementary materials.
**Results, Line 205**	Maintain decimal consistency: "60% (585)" → "6.0% (585)."	Corrected throughout.

**Results, Line 210–211**	"Nutritional status was largely normal 94% (9,214)" → "94.0% (n = 9,214) of children were classified as having normal nutritional status."	Revised.
**Results, Line 218**	Avoid redundancy: "Weighted case = 817 children."	Simplified.
**Results, Line 221**	Clarify higher prevalence among educated mothers.	Added discussion on potential reporting bias and statistical significance.
**Results, Line 243–244**	Add threshold for Gi* statistic.	Threshold added: Z-score > 1.96, $p < 0.05$.
**Results, Line 275**	Explain RR = 2.79.	Interpretation added: children in the cluster nearly three times more likely to experience diarrhea than those outside the cluster.
**Discussion, Line 289**	"shows a slight decline" → "declined modestly."	Revised.
**Discussion, Line 334**	"considerable clusters" → "statistically significant high-risk clusters."	Revised.
**Discussion, Line 353–355**	Clarify higher diarrhea in more educated mothers.	Expanded discussion, referencing reporting bias and caregiver awareness.
**Discussion, Line 359**	Review caregiver reporting in DHS.	Addressed.
**Discussion, Line 367**	"having improved toilets" → "greater access to improved sanitation."	Revised.
**Discussion, Line 388**	Avoid vague phrases like "unmeasured factors."	Replaced with "factors not captured in the DHS data."
**Conclusion, Line 395**	Clarify "maternal support systems."	Revised: "maternal support systems, including caregiving practices and health-seeking behaviors."
**References, Lines 434–516**	Various formatting and errors.	Corrected all reference formatting issues, including WHO, USAID, CDC, merged references, and publication details.
**Figure 3 up to Figure 5**	Figures 3, 4, and 5 may contain copyrighted map images, which are not permitted under the CC BY 4.0 license.	We thank the editor for this comment. Figures 3–5 were created by the authors using **open-access, non-proprietary data**. Administrative boundary shapefiles were obtained from the **Humanitarian Data Exchange (HDX): Mozambique Subnational Administrative Boundaries**, and DHS cluster GPS data were used. No

		proprietary base maps or satellite imagery were used. Figure captions and the Methods section have been updated to confirm CC BY 4.0 compliance.
**Data Availability**	Please provide a complete Data Availability Statement in the submission form, including access information or a justification if the data cannot be made freely accessible.	The DHS data underlying this study are publicly available from the DHS Program website upon registration and approval. Administrative boundary shapefiles were obtained from the Humanitarian Data Exchange (HDX) and are openly accessible

Note: We sincerely thank the editor and reviewers for their constructive feedback. The suggested revisions have improved the clarity, readability, and scientific rigor of the manuscript.

Sincerely,
Thomas Kidanemariam Yewodiaw, on behalf of the authors
