# Supplementary material for: Spatial disparities and multilevel determinants of childhood diarrhea in Mozambique: Evidence from the 2022–2023 Demographic and Health Survey (DHS)
Source: PLoS One. 2026 May 26;21(5):e0330240. doi: 10.1371/journal.pone.0330240 (PMC13210273; doi:10.1371/journal.pone.0330240)
Supplement: Attachment — Submitted filename: Response to Reviewers.pdf [file pone.0330240.s004.pdf]

Response to Reviewers

Manuscript ID: PONE-D-25-40831R2

Title: Spatial Disparities and Multilevel Determinants of Childhood Diarrhea in Mozambique:

Evidence from the 2022–2023 Demographic and Health Survey (DHS)

We sincerely thank the reviewers for their thoughtful and constructive feedback. We greatly appreciate the time and effort taken to provide detailed comments, which have helped us improve the clarity, quality, and presentation of our manuscript. Below is a detailed, point-by-point response.

Reviewer #1 Comments & Authors' Responses

Comment 1: Abbreviations in the abstract should be defined at first use and applied consistently.
Response: We thank the reviewer for highlighting this important point. All abbreviations in the abstract and manuscript have now been defined at first use and applied consistently (e.g., "Demographic and Health Survey (DHS)" is defined at first mention). We appreciate this suggestion, which has improved clarity for readers.

Comment 2: Some formatting inconsistencies persist (spacing between words and reference numbers, lines 415, 430, 434).
Response: Thank you for pointing this out. We have carefully reviewed the manuscript and corrected all spacing and formatting inconsistencies, including the lines mentioned. References now fully follow PLOS ONE style, ensuring consistency throughout the text.

Comment 3: Terminology inconsistencies, e.g., "hotspot" vs "hot spot".
Response: We appreciate the reviewer bringing this to our attention. The term **"hotspot"** has now been standardized consistently throughout the manuscript, including text, tables, and figures.

Comment 4: Several sentences remain lengthy and could be further refined for clarity and conciseness.
Response: Thank you for this valuable observation. We have revised long sentences across the manuscript for improved readability and clarity. Complex sentences were restructured or split into shorter ones to enhance comprehension.

Comment 5: The discussion of reporting bias is somewhat repetitive and would benefit from streamlining.

Response: We are grateful for this suggestion. The discussion section has been streamlined to reduce repetition while maintaining emphasis on reporting bias and its potential effects.

Comment 6: A final thorough proofreading is recommended to ensure overall consistency throughout the manuscript.
Response: Thank you for recommending a final check. The manuscript has undergone full proofreading. Minor typographical and grammatical errors have been corrected, and consistency in abbreviations, terminology, and formatting has been ensured.

Reviewer #2 Comments & Authors' Responses

Comment: All comments have been addressed.
Response: We sincerely thank the reviewer for their confirmation and positive evaluation of our revised manuscript. We greatly appreciate the time and effort taken to review our work, and we are pleased that our revisions met the reviewer's expectations.

We would like to express our sincere gratitude to both reviewers for their constructive and insightful feedback. Their comments have significantly contributed to improving the clarity, rigor, and overall quality of our manuscript. We hope that the revised version meets the journal's standards and expectations.

With regards

Thomas Kidanemariam Yewodiaw (MPH in Epidemiology)
