# Supplementary material for: Spatial disparities and multilevel determinants of childhood diarrhea in Mozambique: Evidence from the 2022–2023 Demographic and Health Survey (DHS)
Source: PLoS One. 2026 May 26;21(5):e0330240. doi: 10.1371/journal.pone.0330240 (PMC13210273; doi:10.1371/journal.pone.0330240)
Supplement: Attachment — Submitted filename: Diarrhea Mozambique.docx [file pone.0330240.s002.docx]

**Line 24:** You said diarrhoea is a major health challenge in Mozambique with regional variations? If sub-national variation is already known what is the significance of this study? Better to rewrite this sentence like “even though significant variations are observed /revealed in previous studies, the exact location where the burden is not well known or up-to-date information is required… ”

**Line 31:** Significant predictors were identified using AORs (95% CI, p ≤ 0.05) rewrite as “statistical significance was determined using p ≤ 0.05 with corresponding AORs”

**Line 33:** “The estimated weighted prevalence of diarrhea in Mozambican children under five was 8.8%” rewrite: “he estimated weighted prevalence of diarrhea in Mozambican under-five children was 8.8%”

**Line 13-132: “**A child is considered to have diarrhea if the mother or caregiver reports that the child passed three or more loose or watery stools in 24 hours within the two weeks preceding the survey” Better to remove “24 hours “within two weeks enough

**Line 250: “**The Global Moran’s I was 0.05, with a Z-score of 1.74 and a p-value of 0.08**”,** but from Figure 1, we can see that the global Moran’s Index is classified as clustered (see the downward dotted line pointing toward clustered), which is supposed to be random. Please revise your data preparation process to settle this contradiction between p-value and z-score, and the Moran’s I picture output from ArcGIS.

**Line 259-260: “**These results suggest that there is moderate but 260 statistically significant clustering of high values across the study area.**” “ *this indicates moderate clustering of high values across the study area***.

**Line 262-263: “score** points to the presence of hotspots—areas where high values of the studied variable are more 263 concentrated than would be expected by chance.**”**

*The studied variable? Which variable? This is the result. Be specific, say diarrhoea*.

Remove the phrase “*by chance*” here

**Line 254 and 265**: You simply said to reveal ----------- it is recommended. Cite the recommendation and reshape your sentence as “to further reveal/uncover---- literature recommends to conduct -----. Therefore, we applied the ----- analysis”

**Line 295:** “*According to the SaTscan analysis*” better to remove and replace the word “According” as Satscan is not specific study or organization like WHO, UNICEF or study done somewhere. “The scan statistics done using Satscan software revealed that….”

**Line 303:** “34.49°E) with a radius of 213.63 km. ” Do you think 213km is suitable to plan an intervention? You can restrict the window size to < 50km, <100km from the very beginning

**Result:** missed the present multilevel regression table and its interpretation if I am not mistaken
